# Volume of blood suctioned during vacuum-assisted breast biopsy predicts later hematoma formation

**DOI:** 10.1186/1756-0500-3-70

**Published:** 2010-03-12

**Authors:** Flora Zagouri, Theodoros N Sergentanis, Philip Domeyer, Dimosthenis Chrysikos, Georgia Giannakopoulou, Nikolaos V Michalopoulos, Panagiotis Safioleas, Ioannis Flessas, Effrosyni Panopoulou, Garifallia Bletsa, George C Zografos

**Affiliations:** 1 Breast Unit, 1st Department of Propaedeutic Surgery, Hippokratio Hospital, University of Athens; 114, Vas Sofias Ave, Athens 116 27, Greece

## Abstract

**Background:**

To evaluate whether the volume of blood suctioned during vacuum-assisted breast biopsy (VABB) is associated with hematoma formation and progression, patient's age and histology of the lesion.

**Findings:**

177 women underwent VABB according to standardized protocol. The volume of blood suctioned and hematoma formation were noted at the end of the procedure, as did the subsequent development and progression of hematoma. First- and second-order logistic regression was performed, where appropriate. Cases with hematoma presented with greater volume of blood suctioned (63.8 ± 44.7 cc vs. 17.2 ± 32.9 cc; p < 0.001, Mann-Whitney-Wilcoxon test for independent samples, MWW); the likelihood of hematoma formation was increasing till a volume equal to 82.6 cc, at which the second-order approach predicts a maximum. The volume of blood suctioned was positively associated with the duration of the procedure (Spearman's rho = 0.417, p < 0.001); accordingly, hematoma formation was also positively associated with the latter (p = 0.004, MWW). The volume of blood suctioned was not associated with patients' age, menopausal status and histopathological diagnosis.

**Conclusion:**

The likelihood of hematoma is increasing along with increasing amount of blood suctioned, reaching a plateau approximately at 80 cc of blood lost.

## Findings

The use of stereotactic vacuum breast biopsy (VABB) is increasing in clinical practice for the assessment of non-palpable mammographic lesions [1-3]. Taking a larger volume of tissue than a standard core biopsy, VABB seems capable of "forgiving" minor targeting inaccuracies [1-3]. That said, it is not without complication, its main one being hematoma formation [1]. Given that VABB is performed in the outpatient setting and women are discharged within one hour after biopsy, the suspicion for hematoma formation, as well as its progression, seems crucial. Worthy to mention is that neither the volume of blood suctioned and collected in the device's bin nor its implications have ever been studied in detail.

The objective of this study is to evaluate whether the volume of blood suctioned during VABB is associated with hematoma formation and progression, patient's age and histology of the lesion. To our knowledge, this is the first study to evaluate the bleeding during VABB and its implications.

## Materials and methods

### Study design, performance of VABB and follow-up of patients

This study enrolled 177 women with a median age of 51 (range 32-81) years, (mean ± SD: 52.4 ± 9.6), who underwent VABB for non-palpable mammographic lesions (BI-RADS 4). Exclusion criteria were: current use of aspirin or non-steroid anti-inflammatory drugs, oral anticoagulants, vitamin E, herbal supplements that may contribute to bleeding and history of bleeding disorders.

VABB was performed on the digital prone table (Mammotest, Fischer Imaging, Denver, CO, USA) using 11-gauge Mammotome vacuum probes, under local anesthesia, according to standardized protocol; 24-96 cores were obtained, according to the results of a double blind study [3]. All procedures were performed by the same surgeon, in the same unit. For local anesthesia in VABB, the two-step approach was adopted: 5 cm^3 ^1% lidocaine without epinephrine (superficial), and 10 cm^3 ^1% lidocaine with epinephrine (deep) were administered [4]. After VABB, mammograms were obtained from the women's breast, as well as from the specimens excised, confirming the successful sampling of the suspicious lesion.

The volume of blood suctioned into the device's bin was registered at the end of the procedure in all cases; importantly, no rinsing saline fluid has been pushed into the VABB probe, as this could have interfered with the measured blood aspiration volume. Hematoma formation was noted after the examination of all traditional mammogram projections (CC, MLO) independently by two radiologists (GG and DK). Compression bandages were applied so as to prevent hematoma. In all cases, the occurrence of clinically significant (i.e. subsequently organized, diameter larger than 3 cm) hematomas within the subsequent four weeks was followed up by ultrasound and clinical examination of the breast.

Concerning the histopathological evaluation, the type of the lesion was noted, i.e. benign lesion, precursor lesion (atypical ductal hyperplasia or lobular neoplasia) [5,6], carcinoma (in situ or invasive).

### Statistical analysis

For the evaluation of the associations between the volume of blood suctioned/hematoma and the duration of the procedure, age, menopausal status, and histopathological diagnosis, non-parametric tests, i.e. Mann-Whitney-Wilcoxon test for independent samples (MWW) or Spearman's rank correlation coefficient, were implemented. This choice has been made given that non-parametric tests are appropriate for non-normal distributions and that they are not heavily influenced by outliers, since the tests allocate ranks to the observations

Focusing on the blood suctioned-hematoma association, logistic regression was performed. Hematoma formation, treated as a dichotomous variable (0: no, 1: yes), was set as dependent variable, whereas the volume of blood suctioned was set as independent variable. Higher-order associations (second- and third-order were evaluated).

Statistical analysis was performed with STATA 8.0 statistical software (Stata Corporation, College Station, TX, USA).

Informed consent was obtained by all participants in this study. This study has been approved by the local Ethics Committee, in compliance to the Helsinki Declaration.

## Results

The features of the study population are depicted in Table 1. The volume of blood suctioned was positively associated with the duration of the procedure (Spearman's rho = 0.417, p < 0.001); accordingly, hematoma formation was also positively associated with the latter (p = 0.004, MWW). However, the volume of blood suctioned was not associated with patients' age, menopausal status and histopathological diagnosis.

**Table 1:** Features of the study population

Continuous variables	mean ± SD	
Age (years)	51.5 ± 10.1	
Duration of the procedure (min)	39.6 ± 14.6	
Volume of tissue excised (cm^3^)	6.34 ± 2.87	
Blood suctioned during the procedure (cc)	21.5 ± 36.6	
		

**Categorical and ordinal variables**	**N**	**Percentage**

***Menopausal status***		
Premenopausal	80	45.2%
Postmenopausal	97	54.8%
***Histopathological diagnosis***		
Benign	116	65.5%
Precursor lesions & carcinomas	61	34.5%
***Hematoma***		
No	161	91.0%
Yes	16	9.0%

Concerning the association with hematoma formation, cases with hematoma presented with greater volume of blood suctioned (63.8 ± 44.7 cc vs. 17.2 ± 32.9 cc; p < 0.001, MWW). Interestingly, at the logistic regression model with hematoma formation set as the dependent variable, the association with the volume of blood suctioned followed a second-order pattern, i.e.

Both coefficients of the first- and the second-order term were statistically significant (p < 0.001). The above equation yields a predicted maximum at the log-odds scale corresponding to a volume equal to 82.6 cc.

## Discussion

Hematoma formation is associated with greater amount of blood suctioned during VABB. Of special interest, the association between volume of blood lost and hematoma formation follows a quadratic pattern. The probability of hematoma formation seems increasing till a volume equal to 82.6 cc, at which the second-order approach predicts a maximum. Noticeably, the exact value at which the maximum has been demonstrated is rather indicative. At a clinical level, this may imply a plateau reached above approx. 80 cc, where the hematoma probability has already been maximized. Indeed, the surgeon should be alert when this crucial domain is reached, as subsequent hematoma formation may be imminent.

Another significant finding that was demonstrated by our study concerns the duration of the procedure. Longer duration is correlated with greater amount of blood lost and hematoma formation. Longer duration may indicate a more strenuous procedure, characterized by more intense pain and greater blood loss [4].

A limitation of the study that needs to be declared pertains to the number of cores (24 to 96) excised; this is in line with the "extended protocol" of a double-blind study previously published by our team [3]. Indeed, the number of cores seems considerably larger than that in the majority of studies excising 6 to 12 cores [3]. Given that the number of cores has not been analysed as a risk factor in this study, it should be kept in mind that the association between the amount of blood suctioned and hematoma may an indirect one. In other words, larger volume of blood suctioned or longer duration of the procedure may be surrogate markers reflecting the effect of a greater number of excised cores, the latter *per se *predicting increased likelihood for hematoma formation.

To our knowledge, this is the first study that provides insight into the implications of blood suctioned during the procedure. Future studies adopting a quantitative approach on the blood lost, along with other factors putatively modulating the latter, such as depth of the lesion, may provide valuable insight into the strenuousness, adverse effects and complications of VABB.

## Competing interests

The authors declare that they have no competing interests.

## Authors' contributions

All authors have read and approved the manuscript. FZ: conceived the idea, participated in the design of the study and assisted in the writing of the manuscript; TS: participated in the design of the study, performed the statistical analysis and assisted in the writing of the manuscript; FD: participated in the design of the study and evaluated critically the manuscript; DC: performed vacuum-assisted breast biopsy and evaluated critically the manuscript; GG: performed vacuum-assisted breast biopsy and evaluated critically the manuscript; NM: performed vacuum-assisted breast biopsy; PS: performed vacuum-assisted breast biopsy; IF: evaluated critically the manuscript; EP: performed vacuum-assisted breast biopsy; GF: evaluated critically the manuscript; GZ: conceived of the study, participated in its design, performed vacuum-assisted breast biopsy and has given final approval of the version to be published
